# Unfolding the enigma of lamina cribrosa morphometry and its association with glaucoma

**DOI:** 10.12669/pjms.35.6.568

**Published:** 2019

**Authors:** Ayesha Saba, Ambreen Usmani, Qamar Ul Islam, Tahira Assad

**Affiliations:** 1 Dr. Ayesha Saba Naz, MBBS. Senior Lecturer, Department of Anatomy, Bahria University Medical and Dental College, Karachi, Pakistan; 2 Dr. Ambreen Usmani, M. Phil, PhD, Vice Principal, Professor, Department of Anatomy. Bahria University Medical and Dental College, Karachi, Pakistan; 3 Dr. Qamar Ul Islam, FCPS. Associate Professor, Consultant PHACO and Vitreoretinal Surgeon, PNS Shifa Hospital, Karachi, Pakistan; 4 Dr. Tahira Assad, M. Phil, PhD, Associate Professor, Department of Pharmacology, Karachi Institute of Medical Sciences, Pakistan

**Keywords:** Glaucoma, Lamina cribrosa, Lamina cribrosa depth, Lamina cribrosa thickness, Optical coherence tomography, Lamina cribrosa morphometry

## Abstract

Primary open angle glaucoma (POAG) is worldwide prevalent ailment, affecting millions, and leading irreversible cause of blindness. The treatment strategies revolve around one modifiable factor, elevated intraocular pressure (IOP), despite POAG presenting with normal IOP. Emphasis is put forth in recent past detecting structural elements of glaucoma; lamina cribrosa (LC) is found to be a promising prospect. Morphological alterations of LC are implicated as early sign before onset of glaucomatous optic neuropathy (GON). In this review, the authors explored scientific works from 1976 till 2018 through Google, Google Scholar, PubMed, HEC Digital Library, Springerlink, and PakMedinet in four months’ time, extracted structural features of LC, its measurable attributes, fresh innovations employed for in-vivo visualization and clinical signs aiding in establishing diagnosis of glaucoma which will assist as a prophylactic measure against GON. No such work has ever been done in South-East Asia including our country. So LC opens a new horizon for research in Pakistan.

## INTRODUCTION

Sclera, fibrous layer of eye coats posterior five sixths, and is composed of diverse size and shapes of collagen fibers, with haphazard alignment. In contrast, cornea has aligned collagenous framework, hence transparent. Sclera is thinnest at equator of eye where extraocular muscular attachments occur, thickest at back and weakest where optic nerve (ON) exits through it, imparting a sieve-like appearance called lamina cribrosa (LC).^1^ LC of optic nerve head (ONH) is a composite layer with mesh-like appearance due to penetrance of retinal ganglion cell (RGC) axons of retina.^1^ LC is thus a reticulated, porous anatomical part of sclera that plugs-in posterior scleral deficiency.^2^

History of LC considered as main site of RGC damage dates well back.^3^ LC is considered a site for initiation of glaucoma pathology. ONH is defined as region from where RGCs exit eye, through LC via scleral canal.^4^ There are no histologically distinct layers of ONH, studies describe it to be consisting of superficial retinal nerve fiber layer (RNFL), prelaminar tissue (PT), laminar tissue composed of LC itself and retrolaminar layers.^5^-^9^

Glaucoma is a leading cause of irreversible loss of vision worldwide.^9^ Historically glaucoma is defined as elevated intraocular pressure (IOP)> 21mmHg, attributed for obstruction to outflow of aqueous humor (AH) at trabecular meshwork, leading to chronic IOP elevation resulting in damage and paralleling visual field (VF) impairment.^10^ Studies done at population level reveal that about 1/3^rd^ of glaucoma sufferers have normal levels of IOP. Thus, current revised definition can be “progressive, chronic optic neuropathy in adults where IOP and other unknown factors contribute to characteristic acquired atrophy of ON and loss of RGCs”.^11^ POAG is most predominant type, accounting for 3/4^th^ of total burden. This form of ON atrophy is manifested by a cascade of clinical signs summiting at level of ON and whole spectrum is known as GON.

LC imperfections are linked with glaucoma advancement.^12^ LC aberrations, associations with GON and glaucoma progression, typical clinical signs are new interests in the field of research and these buzzing factors the author would be trying to gel in this review.

### Structural features of LC

*Macroarchitecture*: LC is most frail component at posterior part of sclera. Limited data is available on in-vivo 3D microarchitecture of LC. It can be accounted for by limitations in manual segmentation of individual pores and beams of LC, which would prove time consuming and inappropriate in conduction of larger studies.^13^

LC is composed of multiple connective tissue layers, knit in a manner of meshwork to give it a fenestrated appearance for passage of RGC axons.^8^,^14^ Although LC is porous throughout, but pores located at superior and inferior poles are found to be largest, and hence are not robust in offering enough mechanical support to RGC axons.^8^ This elaborates more damage occurring to RGC axons in superior and inferior poles. This creates a visually visible clinical sign, known as laminar dots.^4^

*Microarchitecture*: Quantification and visualization of 3D microarchitecture of LC in-vivo had been found difficult due to lack of an automated analysis tool.^14^ Normal pore area measured in one study was (~1460 and 920 µm^2^) and in another was calculated to be 154 to 6637 μm^2^.^13^ In humans was larger than explored in conventional histological studies. This difference in size can be attributed to tissue shrinkage during histological processing. The area between neighboring pores found out in another study was 2.79µm in humans using advanced optics laser scanning ophthalmoscopy (AO-LSO).^15^ On average, LC pore parameters were found to be larger as compared to macaques and so. On average the pores counted in one study in humans were 18, 23 and 53, pore elongation in humans were analyzed as 2.00 ± 0.75. Variables found out on non-human primate’s studies cannot match the human structural characteristics.

### Morphometric and stereological Changes of LC:

*Lamina cribrosa depth (LCD)*: Recently a finding by Zhao et al. showed anterior LC surface depth augmented with worsening of VF status in younger eyes with high-risk ocular hypertension and early glaucoma.^16^ Posterior LC bowing is unique in glaucoma not found in any other optic neuropathies.^17^ LCD can be defined as distance from Bruch’s membrane opening (BMO) level to anterior LC surface.^18^ ONH cupping is a common clinical finding of glaucoma so it is predictable to get a deep LCD according to progression of disease.^19^ Studies reveal posterior deformation and outward migration of LC in response to chronic elevation of IOP^19^, which can be in localized or generalized manner.

*Lamina cribrosa thickness (LCT)*: Cumulative data regarding linear and logarithmic correlation of glaucoma and LC thickness is available.^20^ Thinness of LC in glaucoma attributes to RGC axons damage.^18^ Morphological changes of LCT are proven to occur with advancements in age and fluctuating IOP levels.^21^ LCT can also be in localized and generalized forms. Relations of it can be found with localized RNFL atrophies, disc hemorrhages (DH), myopic refractive aberrations and normal tension glaucoma diagnosis.^22^,^23^ LCT can be calculated as distance between anterior and posterior boundaries of LC surfaces in direction at right angle to anterior aspect of LC at the measurement point.

It was observed in numerous stereological studies conducted over LC that the altered behavior detected in pore enlargement, LCD and LCT occur differentially more in the vertical axis^24^ due to the presence of less compact connective tissue at these poles of LC.

*Lamina cribrosa curvature index (LCCI)*: LCD takes into account choroidal thickness, leading to biased reading of LCD morphology. This hurdle has led to assessment of LCCI. For measurement of LCCI, first lamina cribrosa width (W) is measured by connecting two points on anterior lamina cribrosa surface (ALCS), perpendicular to BMO reference line. Lamina cribrosa curve depth (LCCD) was calculated as the maximum depth from the width reference line to ALCS. Finally, LCCI can now be determined by LCCD/ W×100.^25^ LCCI is robust in a manner it does not take into account thickness of choroid. LCCI was found to be better discriminator between POAG and eyes of healthy individuals. LCD was found to be larger superiorly^25^, while LCCI measurements did not vary between superior and inferior regions. It is a well-known fact that choroid is thicker in superiorly than inferiorly^26^, and hence greater values of LCD in superior regions despite less bowed LC than inferior region, this highlights the limitation of LCD and significance of LCCI.

**Fig. 1:**
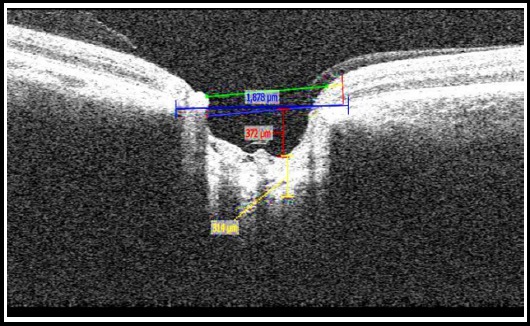
Enhanced depth imaging spectral domain coherence ocular tomography (EDI SD-OCT) measurement of anterior lamina cribrosa depth and lamina cribrosa.

### Innovations in LC visualization

*Optic disc (OD) photography*: was most basic mode to visualize LC. Poor quality of assessment of anterior LC by this technique does not allow for detailed structural LC morphometry, but pore sizes, shapes and numbers can still be ascertained.^27^

### Confocal scanning laser ophthalmoscopy (CSLO)

Used to image ONH in-vivo. CSLO initially was able only to produce 2D images revealing LC pores and its related parameters and ALCS up to some extent, but with advent of adaptive optics (AO), quality of images was improved. AO-CSLO coupled techniques are limited for use in assessment of LC pore morphological characteristics only.^28^

*Optical coherence tomography (OCT)* Developments in image procurement and processing have enabled OCT to gain a unique position in LC imaging. Most initial type of OCT was time-domain (TD-OCT). TD-OCT had restricted axial resolution power (10µm), depth-dependent decrease in sensitivity of capturing image and scattering of light by pigments and blood. Advent of spectral domain ocular computed tomography (SD-OCT) has largely substituted TD-OCT, with axial resolution of up to 2µm, and can capture up to 80,000 A-scans per second.^29^ Recently introduced third generation OCT, known as swept-source (SS-OCT), has a short cavity swept laser with modifiable wavelength instead of diode laser in SD-OCT, that imparts a longer wavelength of 1050nm as compared to 840nm in SD-OCT.^30^ Visualization of deeper ONH structures with improved tissue penetrance is possible. SS-OCT does not require averaging of B-scans due to its raster scanning and excellent sampling density.

Both SD-OCT and SS-OCT have limited use in visualization of deeper ONH owing to attenuated signal strength at greater depths. Current improvements in OCT hardware in form of enhanced depth imaging (EDI) and OCT light-attenuation correction software adaptive compensation (AC) has solved the issue. AC is a post-processing software, helps remove shadows of blood vessels, increases tissue contrast and alters noise over-amplification. EDI-OCT can provide choroidal images and detailed sections of LC. EDI can also be used in conjunction with SS-OCT. AO, newly introduced software^28^, promises for correction in ocular aberrations and improved resolution from 20 to 5 µm.^30^ AO-OCT allows high class images of posterior pole and LC trabecular structure.

Promising new progresses in OCT modality are inventions of micro-OCT with axial resolution of 1µm^31^ and ultrahigh resolution OCT with even sensitive resolution.^32^ There usage advantages are yet to be elucidated.

*Ophthalmoscopic Findings of Glucomatous Optic Neuropathy (GON)*: Optic nerve head (ONH) and optic disc (OD) are used interchangeably despite separate entities. OD is part of ONH examined during ophthalmoscopy.^4^ Researches reveal OD in glaucoma presents with pathognomonic characteristics. The typical features of GON found on slit-lamp biomicroscopy or ophthalmoscopy are:

*OD size and shape*: Average OD diameter vertically is 1.88mm, range 1,700-2,000µm, while horizontal average OD diameter is 1.77mm, range of 1,600-1,800µm. Hence normal healthy OD shape is vertically oval. A small vertical diameter is ≤1.5mm, whereas a large diameter is > 2.2mm.^33^ Mean value for OD surface area is 2.69mm^2^, range of 0.8 mm^2^- 5.54 mm^2^ and shows racial differences.^4^ A small optic cup in small disc suggests GON.^33^

**Table I:** Normal mean values of ophthalmological parameters.

Lamina cribrosa depth LCD (µm)	539.4± 140.5
Lamina cribrosa thickness LCT(µm)	177.7 ± 53.0
Optic disc size (mm^2^)	2.69
CDR	0.4

*Optic cup and cup-to-disc ratio (CDR)*: Optic cup is a centrally located pale area in ONH, having glial tissue, devoid of RGC axons. Pallor accounts for showing collagenous LC element and loss of glial tissue. Studies suggest that area of central pallor matches well with size of cup.^4^ For ascertaining size of optic cup, deviation of blood vessels on ONH surface can be determined (contour method) and is better in comparison for ascertaining it by area of central pallor (color contrast method).^34^

Area of optic cup and disc are interrelated, so small discs have small cup sizes and vice versa.^5^ Average CDR in small disc arises suspicion of GON^4^. Normally optic cup is oval horizontally.^8^ Average CDR value is 0.4, range is from 0.0-0.9. CDR value of 0.4-0.8 indicates physiological cupping or early to moderate glaucoma, CDR of ≥0.8 directs towards GON, unless proven otherwise.^35^ An asymmetrical CDR of 0.2 between eyes of an individual points early stage of glaucoma.^5^ Vertical elongation of optic cup results due to loss of neuroretinal rim (NRR) at superoinferior optic cup poles. Increased CDR with diminished NRR points diffuse RGC loss due to GON.^36^

*Laminar dot sign*: Laminar dot sign occurs due to revelation of LC pores or increased pore size due to loss of RGC axons. Laminar dots are observed in 70% of POAG patients in the shape of a slit. Shape and size of laminar dots signify the glaucomatous visual field loss.^37^

*Optic nerve head hemorrhage (ONHH)*: ONHH occurs seldom in normal retina, but seen frequently in cases of glaucoma, with ONHH appearing at border of OD is hallmark sign of GON.^38^ ONHH is also known as splinter, drance hemorrhages, shaped usually like a flame, located near disc margin or within 1-disc diameter of ONH, excluding the cup. ONHH results due to ischemic changes at the ONH.^8^ They are common in various types of glaucoma, but most preponderant in POAG.^4^ Diminished NRR in progressive stages of glaucoma results in absence of finding of ONHH in such advanced cases.^39^

ONHH arises commonly at locations with NRR notching or RNFL defects. ONHH is seen more commonly in cases of fluctuating IOP. ONHH visualization time spans within 6-10 weeks of onset and associates with RNFL localized lesions and NRR notching.

*Neuroretinal rim (NRR)*: NRR is tissue area between optic cup margin and disc, composed of blood vessels in circumlinear manner. “ISNT” rule is helpful for detection of healthy NRR stating thickness of NRR decreases in order from inferior to superior to nasal to temporal respectively. It corresponds with anatomical placement of optic nerve axons leaving through scleral canal. NRR which does not correspond with ISNT rule and is thin, may be an indicator of GON. NRR always appears “pink” except in terminal glaucoma.^4^ NRR thinning occurs due to generalized or diffuse NRR loss, whereas NRR notching denotes focal NRR loss.

*Parapapillary atrophy (PPA)*: PPA imparts a moth-eaten appearance just outside of outer border of OD, exposing retinal pigment epithelium (RPE), due to degeneration of chorioretinal tissue, imparts irregular pigmentation around OD. Frequency is higher in glaucoma and occurs commonly in conjunction to areas of RNN thinning. PPA appears as peripheral zone alpha (α) and central zone beta (β). Studies relate occurrence of zone beta (β) PPA in glaucoma patients.^40^ Zone beta (β) PPA is linked with progression of glaucoma, with 15% chances of appearance in normal eyes, occurrence is associated with GON.^4^

**Fig. 2:**
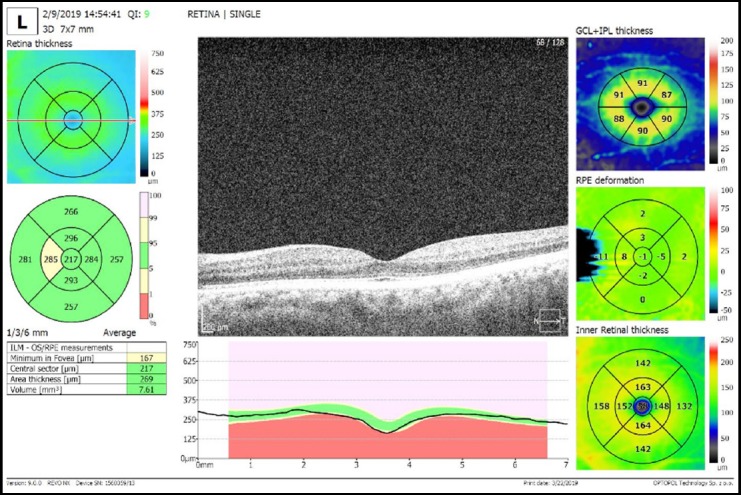
Assessment of sectorial retinal nerve fiber layer thickness using enhanced depth imaging spectral domain ocular coherence tomography, orange turning into red denotes healthy retinal thickness, green turning into blue denotes loss of thickness

*RNFL assessment and detection of its imperfections*: Fine white striations radiating outwards from OD are appearance of normal RNFL, are axons of RGC, enveloped by astrocytes, bounded by Muller cell processes. Most conspicuous in inferotemporal and superotemporal quadrants having thick RNFL distribution, conveying quadrants gleaming appearance. Best way for RNFL visualization is through slit-lamp biomicroscopy or ophthalmoscopy, with bright light and red-free or green light filters. RPE absorbs green light, permits observation of RNFL in dark background. In normal ODs’, bright corrugations of RNFL and indistinctly visible parapapillary vessels are speculated. Obscure parapapillary vessels occur due to overshadowing by healthy RNFL.^4^ RNFL lesions project as loss of striations or presence of dark zones in areas of brightness^4^ with sharpness in detailing of peripapillary vessels. RNFL defects are earliest signs of GON. They occur due to selective destruction in superior and inferior arcuate bundles. Predilection of RNFL damage occurs in sequence of inferior temporal, superior temporal, superior nasal and inferior nasal quadrants. Wedge defects are more common. RNFL loss is 85% specific for glaucoma.

## CONCLUSION

Lamina cribrosa (LC) depth is associated with chronic IOP elevations. It provides substantial clinical findings in evaluation of glaucomatous optic neuropathy (GON), and its detection can allow prophylactic measures before RNFL and VF losses. LCT is also important but measurement of LCCI among all holds a unique position to give a true thickness of LC, excluding choroid. A large optic cup, laminar dot signs with vertical expansion of OD, and asymmetrical CDR ratio of 0.2 in between eyes are not definitive but prognostic features suggestive of GON. High inter-observer variability in estimation of CDR makes diagnosis of GON non reliable. NRR notching, RNFL defects, deviated ISNT rule, ONHH and zone beta (β) PPA have strong associations with primary open angle glaucoma (POAG). Thus detection of above explained fundus features in junction with LC defects in suspicious subjects of glaucoma produce a keystone in the establishment for definitive diagnosis of GON.

### Author`s Contribution:

**ASN** conceived, designed and prepared the manuscript.

**AU and QUI** critically analyzed and reviewed the manuscript.

**TA** edited the manuscript.

## References

[1] Chung HS, Sung KR, Lee JY, Na JH (2016). Lamina cribrosa-related parameters assessed by optical coherence tomography for prediction of future glaucoma progression. Curr Eye Res.

[2] Tan NY, Koh V, Girard MJ, Cheng CY (2018). Imaging of the lamina cribrosa and its role in glaucoma:a review. Exp Ophthalmol.

[3] Quigley H, Anderson DR (1976). The dynamics and location of axonal transport blockade by acute intraocular pressure elevation in primate optic nerve. Invest. Ophthalmol Vis Sci.

[4] Turgut B (2017). Pearls for Correct Assessment of Optic Disc at Glaucoma Diagnosis. US Ophthalmic Rev.

[5] Marjanovic I, Tomas Kubena The Optic Nerve in Glaucoma. The Mystery of Glaucoma.

[6] Susanna Jr R, Medeiros FA, Susanna Jr R, Medeiros FA Ophthalmoscopic aspects of the optic nerve in glaucoma:Normal optic disc. The Optic Nerve in Glaucoma, Rio de Jenerio:Cultura Medica.

[7] Bourne RR (2006). The optic nerve head in glaucoma. Comm Eye Health.

[8] Hayreh SS (2011). Ischemic Optic Neuropathies.

[9] Quigley HA, Broman AT (2006). The number of people with glaucoma worldwide in 2010 and 2020. Br J Ophthalmol.

[10] Tsai JC, Forbes M (2009). Medical management of glaucoma. Professional Communications.

[11] Prum BE, Rosenberg LF, Gedde SJ, Mansberger SL, Stein JD, Moroi SE (2016). Primary open-angle glaucoma preferred practice pattern®guidelines. Ophthalmology.

[12] Faridi OS, Park SC, Kabadi R, Su D, De Moraes CG, Liebmann JM (2014). Effect of focal lamina cribrosa defect on glaucomatous visual field progression. Ophtha.

[13] Ivers KM, Li C, Patel N, Sredar N, Luo X, Queener H (2011). Reproducibility of measuring lamina cribrosa pore geometry in human and nonhuman primates with in vivo adaptive optics imaging. Invest Ophthalmol Vis Sci.

[14] Wang Bo, Nevins JE, Nadler Z, Wollstein G, Ishikawa H, Bilonick RA (2014). Reproducibility of in-vivo OCT measured three-dimensional human lamina cribrosa microarchitecture. Invest Ophthalmol Vis Sci.

[15] Ren R, Yang H, Gardiner SK, Fortune B, Hardin C, Demirel S (2014). Anterior lamina cribrosa surface depth, age, and visual field sensitivity in the Portland progression project. Invest Ophthalmol Vis Sci.

[16] Zhao Q, Qian X, Li L, Sun W, Huang S, Liu Z (2014). Effect of elevated intraocular pressure on the thickness changes of cat laminar and prelaminar tissue using optical coherence tomography. Biomed Mater Eng.

[17] Kim M, Bojikian KD, Slabaugh MA, Ding L, Chen PP (2016). Lamina depth and thickness correlate with glaucoma severity. Indian J Ophthalmol.

[18] Yang H, Williams G, Downs JC, Sigal IA, Roberts MD, Thompson H (2011). Posterior (outward) migration of the lamina cribrosa and early cupping in monkey experimental glaucoma. Invest Ophthalmol Vis Sci.

[19] Inoue R, Hangai M, Kotera Y, Nakanishi H, Mori S, Morishita S (2009). Three-dimensional high-speed optical coherence tomography imaging of lamina cribrosa in glaucoma. Ophtha.

[20] Park HY, Kim SI, Park CK (2017). Influence of the lamina cribrosa on the rate of global and localized retinal nerve fiber layer thinning in open-angle glaucoma. Medicine.

[21] Park SC, Hsu AT, Su D (2013). Factors associated with focal lamina cribrosa defects in glaucoma. Invest Ophthalmol Vis Sci.

[22] Tatham AJ, Miki A, Weinreb RN (2014). Defects of thelamina cribrosa in eyes with localized retinal nerve ﬁber layer loss. Ophtha.

[23] Yoshikawa M, Akagi T, Hangai M, Ohashi-Ikeda H, Takayama K, Morooka S (2014). Alterations in the neural and connective tissue components of glaucomatous cupping after glaucoma surgery using swept-source optical coherence tomography. Invest Ophthalmol Vis Sci.

[24] Quigley HA Histology of human glaucoma optic nerve damage compared to clinical findings in the same eyes. InGlaucoma Update II 1983 (pp. 83-87).

[25] Lee KM, Kim TW, Weinreb RN, Lee EJ, Girard MJ, Mari JM (2014). Anterior lamina cribrosa insertion in primary open-angle glaucoma patients and healthy subjects. PLoS One.

[26] Miller KM, Quigley HA (1988). The clinical appearance of the lamina cribrosa as a function of the extent of glaucomatous optic nerve damage. Ophtha.

[27] Akagi T, Hangai M, Takayama K, Nonaka A, Ooto S, Yoshimura N (2012). In Vivo Imaging of Lamina Cribrosa Pores by Adaptive Optics Scanning Laser Ophthalmoscopy. Invest Ophthalmol Vis Sci.

[28] Akagi T, Hangai M, Takayama K, Nonaka A, Ooto S, Yoshimura N (2012). In vivo imaging of lamina cribrosa pores by adaptive optics scanning laser ophthalmoscopy. Invest Ophthalmol Vis Sci.

[29] Nadler Z, Wang B, Wollstein G, Nevins JE, Ishikawa H, Billonick R (2014). Repeatability of in vivo 3D lamina cribrosa microarchitecture using adaptive optics spectral domain optical coherence tomography. Biomed Opt Express.

[30] Liu L, Gardecki JA, Nadkarni SK, Toussaint JD, Yagi Y, Bouma BE (2011). Imaging the subcellular structure of human coronary atherosclerosis using micro-optical coherence tomography. Nat Med.

[31] Dubois A, Grieve K, Moneron G, Lecaque R, Vabre L, Boccara C (2004). Ultrahigh-resolution full-field optical coherence tomography. Appl Opt.

[32] Kampougeris G, Spyropoulos D, Mitropoulou A, Zografou A, Kosmedis P (2013). Peripapillary retinal nerve fibre layer thickness measurement with SD-OCT in normal and glaucomatous eyes:distribution and correlation with age. Int J Ophthalmol.

[33] Reis ASC, Toren A, Nicolela MT (2012;). Clinical optic disc evaluation in glaucoma. European Ophthalmic Review.

[34] Gandhi M, Dubey S (2013). Evaluation of the optic nerve head in glaucoma. J Curr Glaucoma Pract.

[35] Mackenzie PJ, Mikelberg FS (2009). Evaluating optic nerve damage:pearls and pitfalls. Open Ophthalmol J.

[36] Abe RY, Gracitelli CPB, Diniz-Filho A, Tatham AJ, Medeiros FA (2015). Lamina Cribrosa in Glaucoma:Diagnosis and Monitoring. Curr Ophthalmol Rep.

[37] Moraes D, Gustavo C, Liebmann, Jeffery M, Robert R (2012). Predictive factors within the optic nerve complex for glaucoma progression:disc hemorrhage and parapapillary atrophy. Asia Pacific J Ophthalmol.

[38] Drance S, Anderson DR, Schulzer M (2001). Collaborative Normal-Tension Glaucoma Study Group, Risk factors for progression of visual field abnormalities in normal-tension glaucoma. Am J Ophthalmol.

[39] Bengtsson B, Leske MC, Yang Z, Heijl A, EMGT Group (2008). Disc hemorrhages and treatment in the early manifest glaucoma trial. Ophtha.

[40] Curico CA, Saunders PL, Younger PW, Malek G (2000). Peripapillary Chorioretinal Atrophy:Bruch's Membrane Changes and Photoreceptor Loss. Ophtha.

